# Climate change versus deforestation: Implications for tree species distribution in the dry forests of southern Ecuador

**DOI:** 10.1371/journal.pone.0190092

**Published:** 2017-12-21

**Authors:** Carlos E. Manchego, Patrick Hildebrandt, Jorge Cueva, Carlos Iván Espinosa, Bernd Stimm, Sven Günter

**Affiliations:** 1 Research Department Ecology and Ecosystem Management, Technical University of Munich, Freising, Germany; 2 Departamento de Ciencias Naturales, Universidad Técnica Particular de Loja, Loja, Ecuador; 3 Section Forestry Worldwide, Thünen Institute of International Forestry and Forest Economics, Hamburg, Germany; Chinese Academy of Forestry, CHINA

## Abstract

Seasonally dry forests in the neotropics are heavily threatened by a combination of human disturbances and climate change; however, the severity of these threats is seldom contrasted. This study aims to quantify and compare the effects of deforestation and climate change on the natural spatial ranges of 17 characteristic tree species of southern Ecuador dry deciduous forests, which are heavily fragmented and support high levels of endemism as part of the Tumbesian ecoregion. We used 660 plant records to generate species distribution models and land-cover data to project species ranges for two time frames: a simulated deforestation scenario from 2008 to 2014 with native forest to anthropogenic land-use conversion, and an extreme climate change scenario (CCSM4.0, RCP 8.5) for 2050, which assumed zero change from human activities. To assess both potential threats, we compared the estimated annual rates of species loss (i.e., range shifts) affecting each species. Deforestation loss for all species averaged approximately 71 km^2^/year, while potential climate-attributed loss was almost 21 km^2^/year. Moreover, annual area loss rates due to deforestation were significantly higher than those attributed to climate-change (P < 0.01). However, projections into the future scenario show evidence of diverging displacement patterns, indicating the potential formation of novel ecosystems, which is consistent with other species assemblage predictions as result of climate change. Furthermore, we provide recommendations for management and conservation, prioritizing the most threatened species such as *Albizia multiflora*, *Ceiba trichistandra*, and *Cochlospermum vitifolium*.

## Introduction

Seasonally dry tropical ecosystems have harbored humans for thousands of years. The Americas are no exception because these ecosystems have historically been the preferred zones for settlement and agriculture [1–3]. Due to these and other anthropogenic influences, neotropical seasonally dry forests are the most threatened tropical forests in the world [4], which similarly to other dry areas, could be at risk of degradation due to the effects of climate change [5]. The latest estimates indicate that two-thirds of original neotropical dry forest has been converted to other types of land uses [6]. Some authors argue that the combination of anthropogenic pressure, variability in climatic conditions, and climate change makes tropical dry forests particularly vulnerable regions [4,7]. Of these threats, climate change is perhaps the greatest uncertainty as it might cause species extinctions, range shifts, and biodiversity loss [8–10], particularly in areas where the magnitude of the threats have not been explored yet.

Ecuador, one of the ten most biodiversity-rich nations in the world [11,12], has one-sixth of its territory covered by deciduous and semi-deciduous forests [13] with a reported national deforestation rate of approximately 30 km^2^/year during 2008–2014 [14]. A high proportion of Ecuador’s seasonally dry forests is located in the southwestern part of the country, situated in the Tumbes–Chocó–Magdalena region and adjacent to the Tropical Andes, two large biodiversity hotspots with great species diversity and high levels of species endemism, but also with high habitat loss caused by land-use change [15]. Moreover, these dry forests are particularly susceptible because they are highly fragmented, less than 2.3% of their areas are represented in natural reserves [6], and almost all major conservation threats are linked to habitat degradation [16]. Moreover, these forests not only provide timber and non-timber forest products, but also key ecosystem services such as water flux balance and erosion prevention.

In addition, deforestation rates for the seasonally dry forests of southwestern Ecuador were approximately 29.2 and 57.2 km^2^/year from 1976 to 1989 and 1989 to 2008 [17], respectively, where the most prominent native forest conversions were toward pasture or crops [18,19]. On this subject, there is high certainty that land-use change contributes to environmental degradation and exacerbates the negative impacts of climate change [20]. For instance, temperature increases of 0.1°C to 0.2°C per decade and precipitation variations of 4% per decade have already been detected in Ecuador between 1961 and1990 [21]. In the case of southern Ecuador, Peters et al. [22] found a similar warming pattern of 0.13°C per decade and weak but significant trends in increasing rainfall. In addition, future climate projections for southwestern Ecuador predict a 2°C to 5°C increase in air temperature and a 10% to 40% increase in precipitation by the end of the century [23,24]. Regardless of the future climate scenario, most projections indicate an increase in temperatures and a variation in precipitation values, suggesting precipitation increases in southwestern Ecuador by the end of the century [20].

Altogether, climate change simulations signal an increase in seasonality by 2030 in the areas proximal to the Andes [25], and the effects on native tree species are already being manifested as upslope range shifts [26,27]. However, given that individual species are expected to have different range shifts depending on internal and external traits [28], quantifying the magnitude and direction of these shifts is important in assessing whether the current species composition will remain constant or disaggregate with future changes. Convergent changes could indicate that ecosystem compositions will remain stable (assuming equal displacement ability) while divergent patterns may indicate new ecosystem compositions, with unknown consequences on synecology, ecosystem functions, and thus ecosystem services. In this regard, a meaningful approach using a response-and-effect functional framework was suggested by Suding et al. [29] to minimize these uncertainties.

Furthermore, for efficient planning, implementation of conservation measures, and sustainable land use, prioritizing efforts according to threats and vulnerabilities is important. Therefore, it is critical to differentiate between potential climate change and deforestation threats, identifying patterns at both species and community levels. In this study, we use species distribution models to estimate and compare potential climate change threats with current deforestation patterns for a characteristic plant community of 17 tree species in the seasonally dry forest of Ecuador. We hypothesize that (a) deforestation differ in magnitude and spatial distribution from potential range shifts due to climate change; (b) both patterns do not exhibit species-specific effects; and (c) individual species responses reveal a convergent pattern, maintaining community structure. In this study, we aim to provide a scientific reference frame to identify the lesser of two evils and provide a basis for effective resource allocation in forest conservation and sustainable land use.

## Materials and methods

### Study area

Although the precise geographical extent of the dry deciduous forest region in Ecuador lacks unanimous consensus [30–35], authors agree on the presence of several distinct ecosystems within this region. In this study, we focus on the dry deciduous forest on hillsides of southwestern Ecuador, as proposed and described by Aguirre et al. [36], because this ecological unit is heavily threatened by human intervention [17,37,38]. The locality is characterized by a 5-month dry season, mean annual temperature of 20°C–26°C, precipitation ranging from 300 to 700 mm/year [39], and a high number of endemic species [40,41].

### Species records

According to the criteria of Aguirre et al. [36], we selected all characteristic tree species of the dry deciduous forest on hillsides of southwestern Ecuador, excluding predominantly shrub life-forms [42,43] (Table 1). All 17 tree species are used as local timber or other wood products [43], and although these species are categorized as distinctive of the area, they do not exclusively occur in this region [44]. Presence records were obtained from the GBIF database [45] and complemented with inventory data from our permanent plots as well as herbarium records at the Universidad Nacional de Loja (S1 Dataset).

**Table 1:** Characteristic species of the dry deciduous forest on hillsides of southwestern Ecuador.

Selected species	Records	Synonyms	Family	Elevation
*Albizia multiflora* (Kunth) Barneby & J.W. Grimes	51	*Acacia multiflora; Pithecellobium multiflorum*	Mimosaceae	0–1000
*Bursera graveolens* (Kunth) Triana & Planch.	42	*Elaphrium graveolens; Spondias edmonstonei*	Burseraceae	0–2000
*Caesalpinia glabrata* Kunth	65	*Caesalpinia paipai; Caesalpinia corymbosa*	Caesalpiniaceae	0–500
*Cavanillesia platanifolia* (Bonpl.) Kunth	07	*Pourretia platanifolia*	Malvaceae	0–500
*Ceiba trichistandra* (A. Gray) Bakh	39	*Eriodendron trichistandrum*	Malvaceae	0–500
*Chloroleucon mangense* (Jack.) Britton & Rose	53	*Pithecellobium mangense; Mimosa mangensis*	Mimosaceae	0–1000
*Cordia macrantha* Chodat	17	*-*	Boraginaceae	0–500
*Coccoloba ruiziana* Lindau	33	*-*	Polygonaceae	0–1000
*Colicodendron scabridum* (Kunth) Seem	26	*Capparis scabrida*	Capparaceae	0–500; 1000–2000
*Cochlospermum vitifolium* (Willd.) Spreng.	40	*Bombax vitifolium*	Cochlospermaceae	0–1000
*Erythrina velutina* Willd.	23	*Erythrina splendida*	Fabaceae	0–500
*Geoffroea spinosa* Jacq.	51	*Geoffroea striata; Robinia striata*	Fabaceae	0–500
*Guazuma ulmifolia* Lam.	52	*-*	Sterculiaceae	0–2500
*Handroanthus chrysanthus* (Jacq.) S.O. Grose	52	*Tabebuia chrysantha*	Bignonaceae	0–1000
*Loxopterygium huasango* Spruce ex Engl.	24	*-*	Anacardiaceae	0–2000
*Piscidia carthagenensis* Jacq.	55	*Piscidia acuminata; Ichthyomethia acuminate*	Fabaceae	0–500
*Prosopis juliflora* (Sw.) DC.	30	*Mimosa juliflora*	Mimosaceae	0–500

Tree species and number of occurrence records used to produce the species distribution models, along with register data from the Catalogue of the Vascular Plants of Ecuador [42] that were validated through The Plant List [46]. Elevation is given in m a.s.l.

The geographical accuracy of species records was ensured by validating metadata and verifying individual coordinates through the OpenLayers plugin 1.1.4 for QGIS. Then, we used the R script ElimCellDups [47] to retain a single species occurrence per raster cell.

### Predictor variables

**Fig 1:**
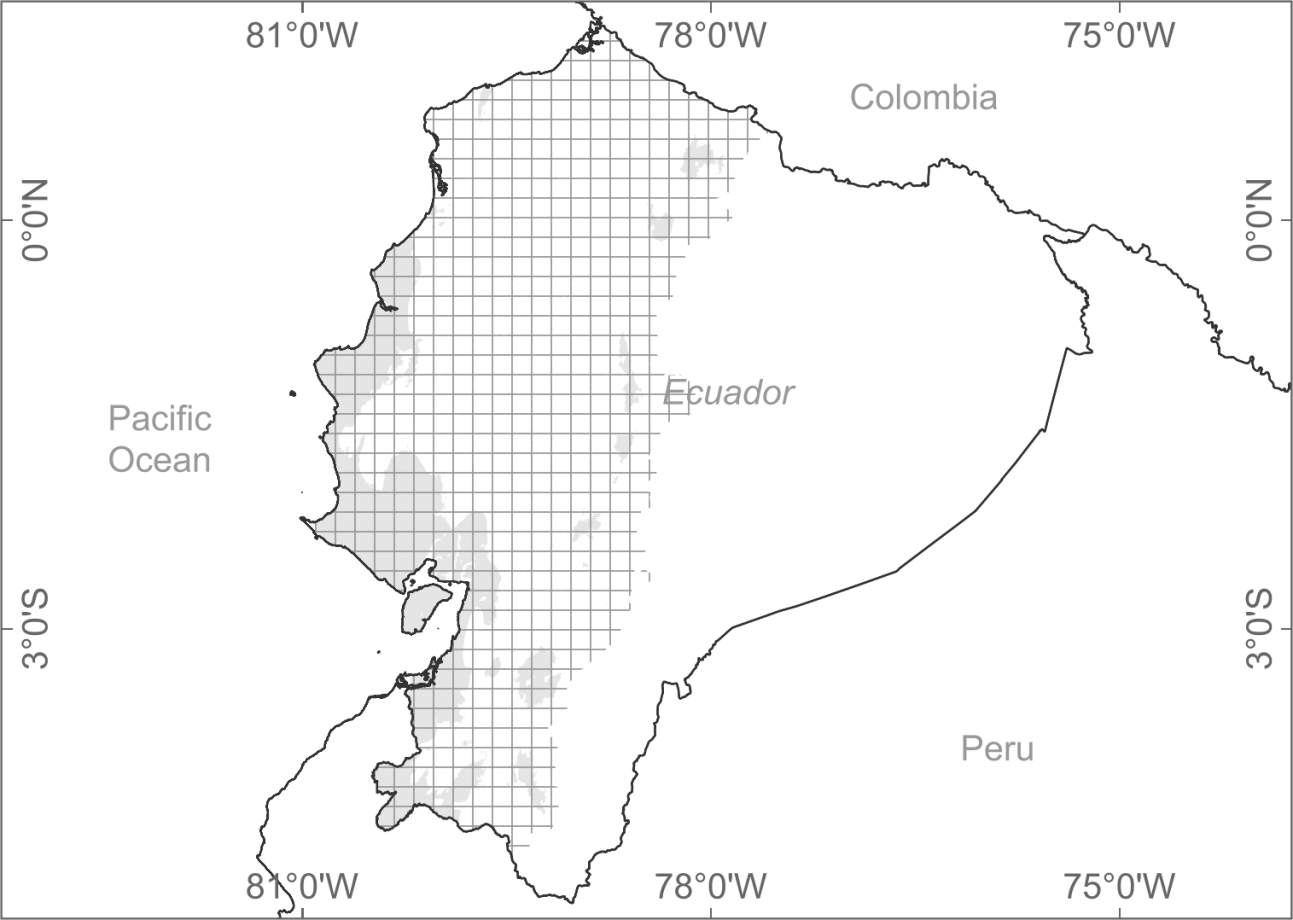
Environmental space extent during modeling. Gridded area represents the geographical space, (i.e., input data) used to run the models. Provinces located in the Amazon region were excluded as well as major islands. Shaded gray area illustrates the bioclimatic demarcation of xeric environments from the Ministry of Environment of Ecuador [13], which to the West encompasses different forests types, including the totality of the Ecuadorian dry forests and a fraction of the Tumbes–Piura dry forests.

Present and future bioclimatic layers were obtained from WorldClim.org [48] at 30-arc second resolution, approximately 1 × 1 km near the equator. In addition, the following three topographical variables were used: soil classification based on the USDA denominations; absolute depth to bedrock; and soil organic carbon stock. All three variables were obtained from SoilGrids.org [49–51], and their grid resolutions were adjusted to match the bioclimatic layers. The chosen future scenario was the most extreme possible outcome for 2050 and utilized the Representative Concentration Pathway (RCP) 8.5 from the global circulation model CCSM4.0 in accordance with the fifth intergovernmental panel on climate change assessment report [52]. The complete list of variables considered in the models is shown in Table 2. Furthermore, to account for the fundamental role of environmental space during modeling [53,54], we delimited the spatial grid coverage to the Coastal and Andean regions of Ecuador, excluding major islands and Amazon region provinces (Fig 1).

**Table 2:** Environmental variables considered in species distribution modeling.

Variable	Code	Data resolution
Annual mean temperature [°C]	bio01	~1000 ^a^
Mean diurnal range (Mean of monthly (max temp–min temp)) [°C]	bio02	~1000 ^a^
Isothermality ((bio2/bio7) × 100) [%]	bio03	~1000 ^a^
Temperature seasonality (standard deviation × 100) [°C]	bio04	~1000 ^a^
Max. temperature of warmest month [°C]	bio05	~1000 ^a^
Min. temperature of coldest month [°C]	bio06	~1000 ^a^
Temperature annual range (Bio5-Bio6) [°C]	bio07	~1000 ^a^
Mean temperature of wettest quarter [°C]	bio08	~1000 ^a^
Mean temperature of driest quarter [°C]	bio09	~1000 ^a^
Mean temperature of warmest quarter [°C]	bio10	~1000 ^a^
Mean temperature of coldest quarter [°C]	bio11	~1000 ^a^
Annual precipitation [mm]	bio12	~1000 ^a^
Precipitation of wettest month [mm]	bio13	~1000 ^a^
Precipitation of driest month [mm]	bio14	~1000 ^a^
Precipitation seasonality (coefficient of variation) [%]	bio15	~1000 ^a^
Precipitation of wettest quarter [mm]	bio16	~1000 ^a^
Precipitation of driest quarter [mm]	bio17	~1000 ^a^
Precipitation of warmest quarter [mm]	bio18	~1000 ^a^
Precipitation of coldest quarter [mm]	bio19	~1000 ^a^
Soil classification, TAXOUSDA [predicted most probable class]	Sclass	250 ^b^
Soil depth (absolute depth to bedrock) [cm]	Sdepth	250 ^b^
Soil organic content (fine earth fraction) [g/kg]	Sorgco	250 ^b^

Units indicated inside brackets. Data resolution expressed in m^2^. Sources of information are indicated below:

^a^ Worldclim.org.

^b^ Soilgrids.org.

### Modeling potential species distributions

The environmental niche modeling was produced with Maxent v3.3, a widely used algorithm for assessing species distributions that can rely on presence-only data and retain a strong predictive power compared to other approaches [55]. To obtain biological meaningful outcomes, we followed the recommendations of Merow et al. [56]. To minimize multicollinearity and model overfitting, a principal component analysis (PCA) was performed with all predictor variables and those highly correlated were removed (r^2^ > 0.8). In addition, to account for occurrence record sampling biases, we built a simple biased raster file [56] based on Ecuadorian access roads, where we assigned a 2.5 km buffer around roadways and assumed that the probability of finding records inside this zone was double that of the surrounding area. We programmed the console to run 5 replicates of each model and left the remaining settings at default values.

The outcomes of the environmental niche modeling were converted to binary-type using the 10% training presence threshold as the absence criteria. Analyses of rasters, vectors, and area calculations were performed using QGIS 2.2.0, including the principal component analysis through the python plugin PCA v0.3. Model evaluation was performed by the area under the receiver-operator (AUC) of the receiver operator characteristic (ROC), a debated, but prevalent rank-based metric to assess predicted distribution model accuracy [56, 57]. This metric is the probability that a random presence locality is ranked higher than a random absence location. An AUC value of 0.5 indicates that the prediction is not better than random; < 0.5 is worse than random; 0.5–0.7 indicates poor performance; 0.7–0.9 represents reasonable or moderate performance; and > 0.9 signifies high performance [58].

### Calculation of deforestation and climate change metrics

To calculate the deforestation and climate change metrics affecting each species, we used 2008 and 2014 public land-cover and land-use data from the Ecuadorian Ministry of Environment that is available in digital cartography form [13]. Next, we used forest loss and/or remnant native forest information to mask the binary outcomes of individual species distribution models, for both present and future models. Thus, we obtained species-specific approximations of the affected area for the periods: 2008–2014 and 2014–2050. Given that estimated area loss attributed to deforestation was calculated for a 6-year period and the area threatened by climate change was determined for a 36-year period, we standardized values by calculating annual rates of loss and compared values for all 17 species using a paired t-test.

Distribution area measurements and landscape metrics for each species were computed by the python plugin LecoS v2.0.7 [59], an alternative to the more comprehensive FRAGSTATS, that has the advantage of working within the QGIS processing framework. For this, we transformed the deforestation and climate change masked outputs to raster format using a 250 × 250 m cell size, which set the minimum detectable area for any landscape metric to 0.0625 km^2^. In addition, we also superimposed (i.e., stacked) species distributions in native forests that were unthreatened by climate change, to evaluate how the modeled species overlap differed from the deforestation and climate change threats.

To summarize and visually compare the changes in distribution attributed to deforestation and climate change, we calculated the core distributional shifts (i.e., area centroids) for each species according to two time frames: 2008–2014 and 2014–2050. For this, we used SDMtoolbox [60], a python-based GIS toolkit for automating analyses in ecology and species distribution models. This analysis reduces the distribution area to a single point and creates a line that represents the magnitude and direction of change. In this section, climate change vector lengths were corrected to reflect six years of change to match the deforestation time interval.

## Results

### Species records and predictor variables

Presence records for all 17 species ranged from 7 to 62 unique points per raster cell, with more than two-thirds of all species having > 30 records, generally perceived as an optimal number of locations to generate consistent models [55]. From the pool of 22 predictor variables, eight were selected based on their correlation coefficients and were considered biologically meaningful for dry forest ecosystems (Table 3). The designated variables to produce the models included annual mean temperature, mean diurnal range, precipitation seasonality, wettest quarter precipitation, driest quarter precipitation, soil classification, depth to bedrock, and soil organic content.

**Table 3:** Correlation matrix of predictor variables.

	Temperature											Precipitation								Topography		
	bio01	bio02	bio03	bio04	bio05	bio06	bio07	bio08	bio09	bio10	bio11	bio12	bio13	bio14	bio15	bio16	bio17	bio18	bio19	Sclass	Sdepth	Sorgco
bio01*		0.76	0.88	0.86	0.99	0.98	0.89	1.00	1.00	1.00	1.00	0.80	0.87	0.44	0.79	0.68	0.46	0.82	0.44	0.53	0.75	0.57
bio02*			0.98	0.72	0.90	0.77	0.99	0.85	0.86	0.85	0.86	0.74	0.75	0.48	0.76	0.74	0.50	0.66	0.52	0.50	0.72	0.72
bio03				0.74	0.92	0.80	0.98	0.87	0.88	0.87	0.88	0.79	0.79	0.56	0.81	0.78	0.58	0.71	0.56	0.48	0.75	0.75
bio04					0.86	0.84	0.78	0.87	0.85	0.87	0.85	0.57	0.68	0.26	0.79	0.66	0.26	0.66	0.19	0.55	0.78	0.52
bio05						0.96	0.93	0.99	0.99	0.99	0.99	0.80	0.86	0.45	0.82	0.85	0.47	0.80	0.46	0.54	0.84	0.61
bio06							0.80	0.98	0.98	0.98	0.98	0.78	0.86	0.41	0.85	0.85	0.43	0.82	0.41	0.50	0.84	0.49
bio07								0.88	0.89	0.89	0.88	0.74	0.72	0.46	0.78	0.75	0.48	0.69	0.49	0.52	0.75	0.72
bio08									0.99	1.00	0.99	0.79	0.87	0.43	0.83	0.85	0.45	0.82	0.43	0.53	0.85	0.56
bio09										0.99	1.00	0.80	0.87	0.44	0.82	0.86	0.46	0.82	0.46	0.52	0.85	0.57
bio10											0.99	0.79	0.87	0.43	0.83	0.85	0.45	0.82	0.43	0.53	0.85	0.56
bio11												0.80	0.87	0.44	0.82	0.86	0.46	0.82	0.45	0.52	0.85	0.56
bio12													0.95	0.75	0.62	0.95	0.77	0.93	0.69	0.28	0.74	0.61
bio13														0.55	0.77	0.99	0.56	0.97	0.52	0.36	0.84	0.55
bio14															0.16	0.56	0.99	0.55	0.78	0.08	0.35	0.53
bio15*																0.75	0.17	0.70	0.22	0.56	0.78	0.51
bio16*																	0.57	0.97	0.53	0.35	0.79	0.55
bio17*																		0.56	0.80	0.08	0.36	0.54
bio18																			0.44	0.32	0.82	0.51
bio19																				0.09	0.28	0.50
Sclass*																					0.45	0.33
Sdepth*																						0.58
Sorgco*																						

Highly correlated variables were ignored (correlation > 0.8).

* Selected variables to produce the models included annual mean temperature (bio1), mean diurnal range (bio2), precipitation seasonality (bio15), precipitation of wettest quarter (bio16), precipitation of driest quarter (bio17), soil classification (Sclass), absolute depth to bedrock (Sdepth), and soil organic content (Sorgco).

Model outcomes consistently indicated that the two most important predictor variables were the precipitation of driest quarter and soil classification, continuous and categorical variables, respectively. In contrast, variables that contributed least to the model were precipitation of wettest quarter and precipitation seasonality (Table A in S1 Appendix).

### Species distribution modeling and evaluation

We obtained robust evaluation metrics for 14 species (AUC ≥ 0.90), while the remaining three species had lower AUC values (between 0.79 and 0.88), which are still considered an indicative of reasonable to moderate performance (Table 4).

**Table 4:** Average model AUC values.

Species	AUC	SD
*Albizia multiflora*	0.995	± 0.014
*Bursera graveolen*	0.948	± 0.021
*Caesalpinia glabrata*	0.936	± 0.018
*Cavanillesia platanifolia*	0.981	± 0.011
*Ceiba trichistandra*	0.958	± 0.022
*Chloroleucon mangense*	0.944	± 0.043
*Coccoloba ruiziana*	0.926	± 0.018
*Cochlospermum vitifolium*	0.920	± 0.054
*Colicodendron scabridum*	0.950	± 0.029
*Cordia macrantha*	0.900	± 0.069
*Erythrina velutina*	0.925	± 0.062
*Geoffroea spinosa*	0.926	± 0.041
*Guazuma ulmifolia*	0.797	± 0.019
*Handroanthus chrysanthus*	0.869	± 0.029
*Loxopterygium huasango*	0.965	± 0.031
*Piscidia carthagenensis*	0.933	± 0.029
*Prosopis juliflora*	0.884	± 0.038

AUC, or area under the receiver-operating characteristic (ROC) curve. Values correspond to the mean of 5 model runs.

**Fig 2:**
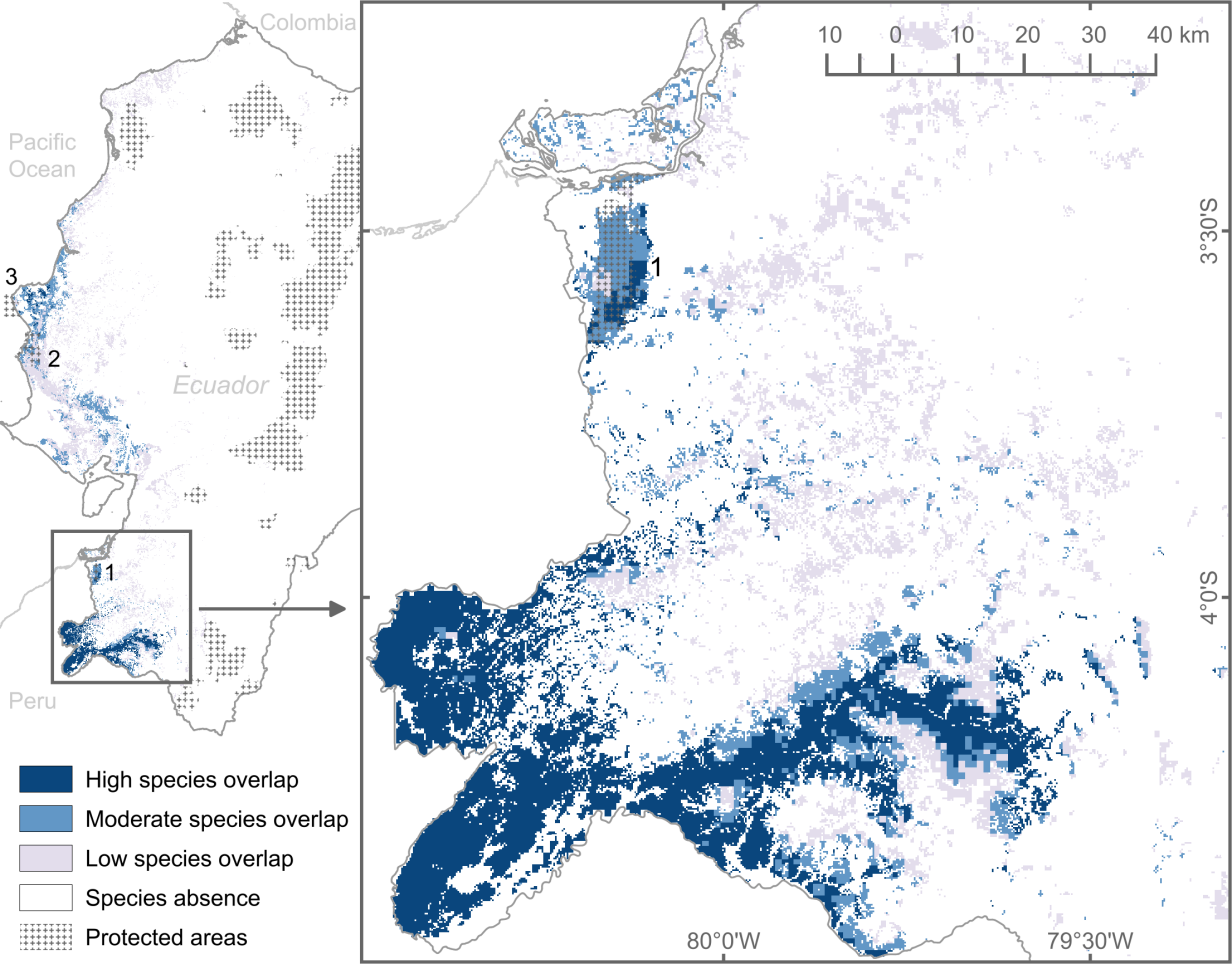
Stacked model projections of 17 characteristic trees species of Ecuadorian dry deciduous forests. Potential species presence was based on 2014 native forest remnants. Low, moderate, and high species overlaps represent 1–6, 7–12, and 13–17 species, respectively. Pattern fill illustrates the natural areas protected by the Ministry of Environment of Ecuador [13], excluding strictly maritime areas, mangroves, and recreational areas, as well as private protected areas. Numbered regions highlight the protected areas within the dry deciduous forest (1. Reserva Ecologica Arenillas, 2. Parque Nacional Machalilla, and 3. Refugio de Vida Silvestre Pacoche). Enlarged image on the right emphasizes potential species overlap in southwestern Ecuador.

In addition, to corroborate the model outcomes, we built a stacked map of all species present in remnant native forests (Fig 2), which revealed a concentration of species distributions in southwestern Ecuador that agreed with ecological descriptions.

### Assessment of deforestation and climate change

**Fig 3:**
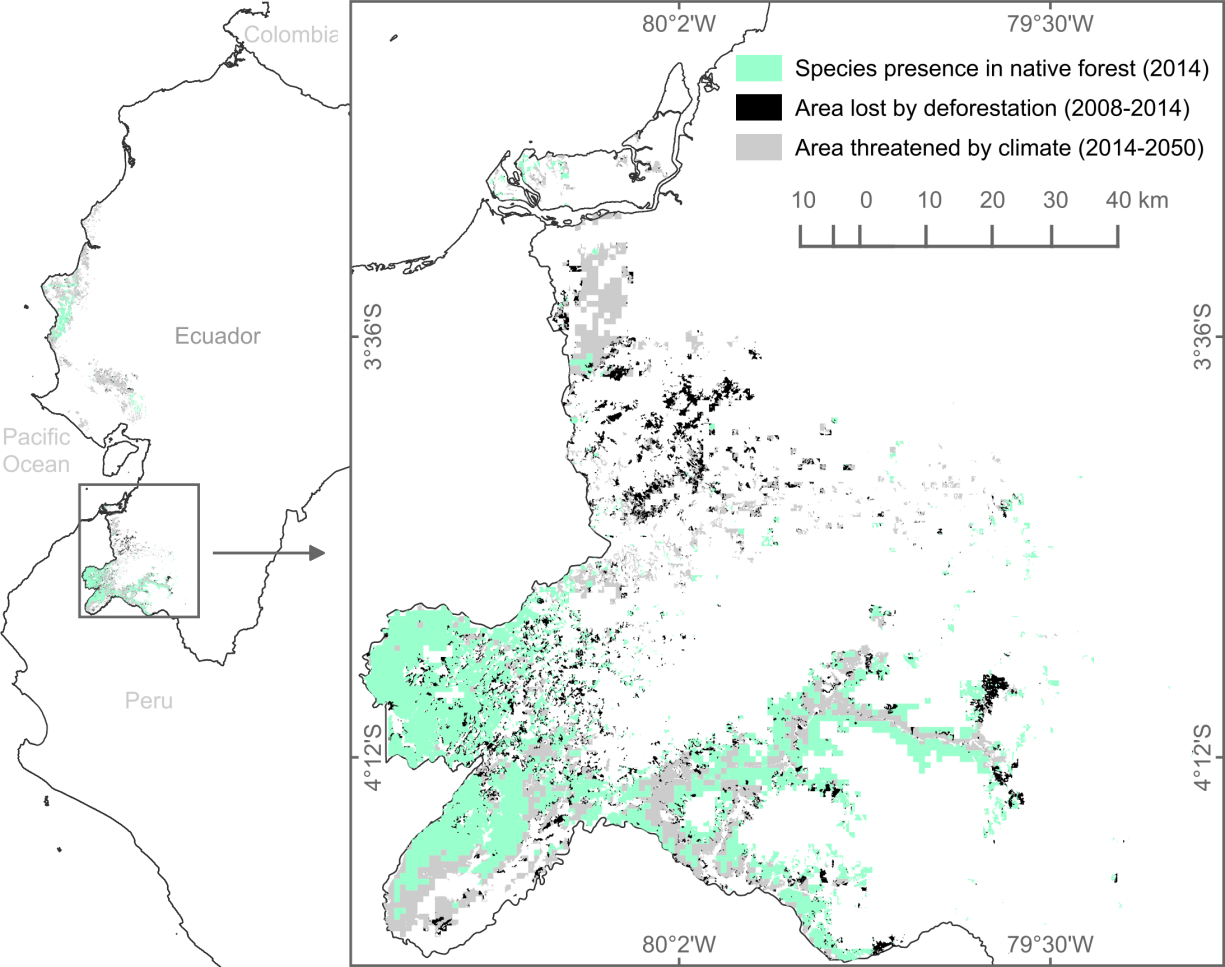
Potential distribution areas for *Albizia multiflora*. The map on the left shows the species model for continental Ecuador, while the enlarged map on the right highlights species presence calculations in southwestern Ecuador. Green, black, and gray colors represent areas of remnant native forest, areas lost to deforestation, and remnant forest areas threatened by climate change, respectively.

Binary potential distributions were combined with land-cover and land-use data to obtain the following three area estimates for each species: area lost by deforestation, remnant native forest area unthreatened by climate change, and area threatened by climate change. An illustration for one species is shown in Fig 3, and maps for all 17 species are listed in Table B in S1 Appendix.

For all 17 tree species except *Cavanillesia platanifolia*, deforestation consistently represents a greater threat in reducing distribution areas compared with climate change (CCSM4.0, RCP 8.5, 2050). Our annual loss estimates from deforestation ranged from approximately 9 to 200 km^2^/year across species, while estimated annual loss from climate change ranged from 4 to 60 km^2^/year. Results from the paired t-test identified a significant difference between the distribution-loss associated with deforestation and the loss attributed to climate change (p-value = 0.001, t = 3.93, and df = 16). Similarly, t-tests for landscape variables also showed significance, particularly for landscape cover (p-value < 0.0001), edge length (p-value = 0.01), number of patches (p-value = 0.0002), and mean patch area (p-value < 0.0001) (Table 5). In summary, there is evidence that all tree species are highly affected by deforestation processes, but there are three species, namely *Albizia multiflora*, *Ceiba trichstandra*, and *Cochlospermum vitifoliu* that might be susceptible to additional climate change pressure, which strongly affects the area and number of patches of these species.

**Table 5:** Annual landscape change attributed to deforestation and climate change.

	Landscape cover (km^2^)***		Proportion (%)***		Edge length (km)*		Number of patches (%)**		Greatest patch area (km^2^)***		Mean patch area (km^2^)***	
	*Deforest*.	*Climate*	*Deforest*.	*Climate*	*Deforest*.	*Climate*	*Deforest*.	*Climate*	*Deforest*.	*Climate*	*Deforest*.	*Climate*
*Albizia multiflora*	−68.68	−48.36	−0.05	−0.03	−45.25	−110.85	+1.7	−0.54	−77.27	−16.79	−0.11	-0.02
*Bursera graveolens*	−51.42	−8.70	−0.04	−0.01	−34.62	−15.58	+1.4	−0.1	−99.68	+6.69	−0.08	−0.005
*Caesalpinia glabrata*	−87.22	+31.76	−0.06	+0.02	-64.75	+95.25	+1.0	+0.6	−101.00	+6.49	−0.07	−0.000
*Cavanillesia platanifolia*	−9.25	−10.93	−0.01	−0.01	+4.67	−23.72	+2.0	−0.6	−3.35	−7.06	−0.14	−0.05
*Ceiba trichistandra*	−42.73	−33.86	−0.03	−0.02	−10.38	−95.34	+2.3	−1.5	−97.98	−21.49	−0.11	+0.01
*Chloroleucon mangense*	−30.23	−10.77	−0.02	−0.01	−13.62	−30.51	+1.1	−0.5	−82.30	−6.97	−0.07	−0.002
*Cordia macrantha*	−113.30	+73.92	−0.08	+0.05	−106.45	+205.03	+2.0	+0.9	−105.82	+1.71	−0.10	+0.01
*Coccoloba ruiziana*	−69.07	+31.56	−0.05	+0.02	−40.45	+74.63	+1.0	+0.3	−102.62	−1.12	−0.07	+0.01
*Colicodendron scabridum*	−43.13	+48.90	−0.03	+0.03	−66.50	+132.69	+1.2	+1.2	−14.47	+19.53	−0.07	+0.01
*Cochlospermum vitifolium*	−138.22	−69.81	−0.09	−0.05	−83.45	−201.47	+2.0	−0.7	−101.17	+3.91	−0.11	−0.01
*Erythrina velutina*	−89.05	−19.29	−0.06	−0.01	−80.55	−24.78	+2.0	0.00	−105.20	+0.88	−0.10	−0.01
*Geoffroea spinosa*	−72.23	−4.32	−0.05	−0.00	−94.38	−7.09	+1.6	−0.05	−101.40	+4.05	−0.08	−0.002
*Guazuma ulmifolia*	−170.70	+65.45	−0.11	+0.04	−164.00	+217.36	+1.0	+0.6	−9.22	+46.94	−0.07	−0.003
*Handroanthus chrysanthus*	−197.15	+22.33	−0.13	+0.01	−148.97	+88.87	+1.4	+0.3	−27.85	+8.74	−0.09	−0.001
*Loxopterygium huasango*	−38.42	−11.09	−0.03	−0.01	−7.53	−27.09	+2.0	−0.7	−99.47	−14.93	−0.12	−0.003
*Piscidia carthagenensis*	−56.55	−14.77	−0.04	−0.01	−38.88	−48.38	+0.4	−0.4	−97.73	−1.08	−0.04	−0.001
*Prosopis juliflora*	−121.00	+49.45	−0.08	+0.03	−57.20	+174.87	+1.0	+1.2	−26.38	+10.62	−0.09	−0.01

Minimum patch area detected for all species was equal to the grid cell size (0.0625 km^2^). The proportion (%) of total area was calculated based on the environmental space used during modeling (approximately 130,000 km^2^). The number of patches depicts the percent change relative to all patches.

Asterisks indicate statistical difference:

(*) represents significance at p-value = 0.01.

(**) indicates high significance at p-value = 0.001.

(***) very high significance at p-value < 0.0001.

In addition, seven species displayed an increase in their future distributions after area gain was accounted for in models, with an estimated area gain ranging from 22 to 74 km^2^/year. These seven species (i.e., *Caesalpinia glabrata*, *Coccoloba ruiziana*, *Colicodendron scabridum*, *Cordia macrantha*, *Guazuma ulmifolia*, *Handroanthus chrysanthus*, and *Prosopis juliflora*) also showed corresponding increases in their total edge lengths and number of patches, assuming recolonization.

Stacked distributions to explore species overlap predictions also suggest that deforestation has a greater effect on annual loss than climate change. For instance, areas with higher species overlap (13 to 17 species) decrease at approximately 34 km^2^/year owing to deforestation, compared to less than 6 km^2^/year owing to the future climate scenario (Table C in S1 Appendix). Moreover, in southwestern Ecuador, deforestation seem to reduce the number of species at all elevations, while the future climate scenario appears to shrink species overlaps primarily at higher altitudes and not in the lowlands, where projections show an increase in the number of overlapping species. In addition, the spatial deforestation hotspot does not coincide with the hotspot of species loss attributed to climate change (Figure D in S1 Appendix).

**Fig 4:**
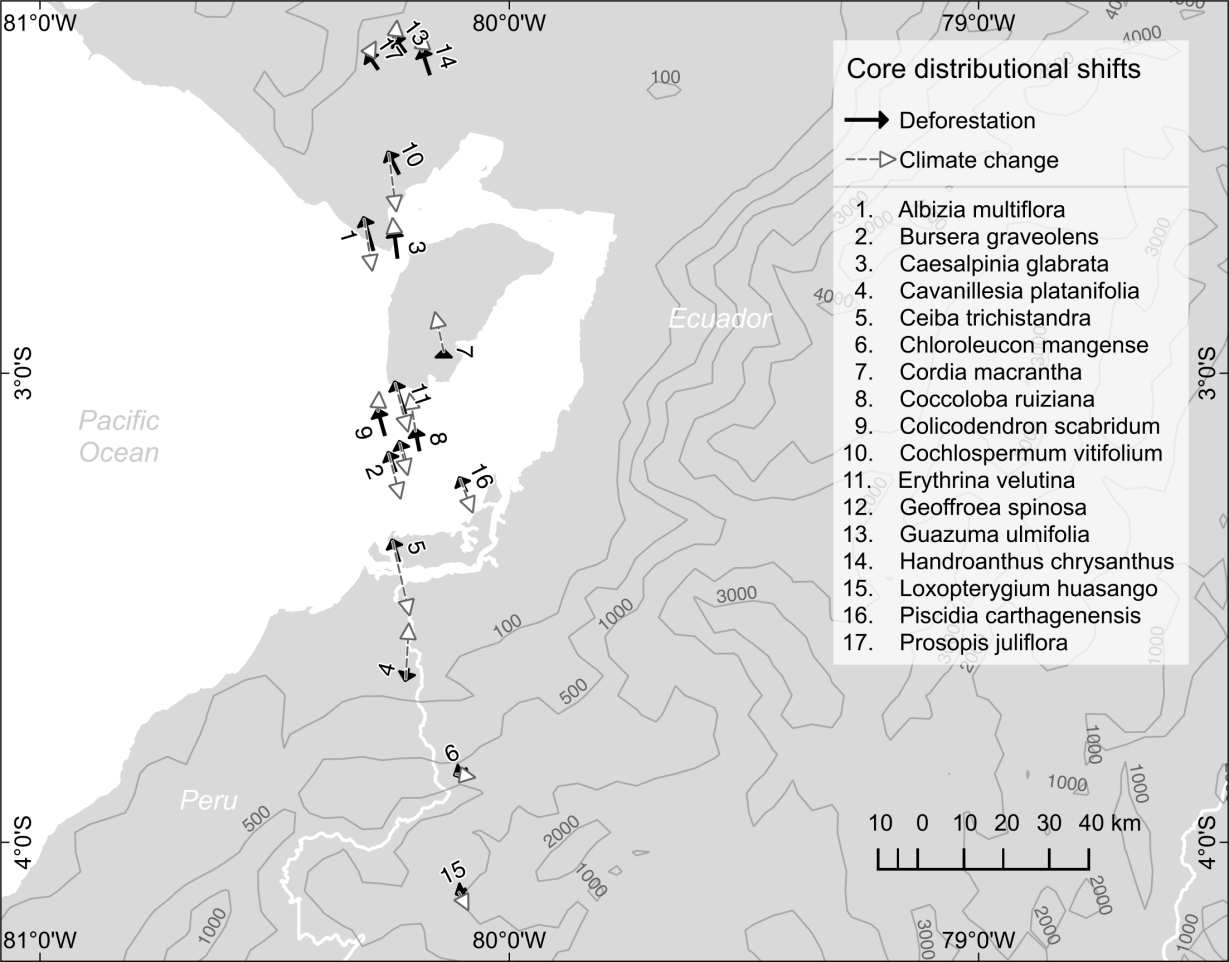
Vectors depicting the core distributional shifts of characteristic tree species in the Ecuadorian dry deciduous forest. Solid black lines show the change in the centroid of distribution after deforestation (2008–2014) and dashed gray lines represent the projected change under the future climate scenario CCSM4.0, RCP 8.5 (2014–2050). Climate change vector lengths were adjusted to show the same time interval as deforestation (i.e., 6 years). White lines denote country borders, and numbers > 100 represent altitude of contour lines in meters.

Finally, based on the centroids of distribution, we produced a summary map showing the core distributional shifts for all species considered in this study (Fig 4). From this map, deforestation appears to push the majority of species distributions northward at a mean rate of 0.8 km/year. However, the climate change projections result in a set of species headed southward at a mean rate of 1.4 km/year (i.e., *Albizia multiflora*, *Cochlospermum vitifolium*, *Erythrina velutina*, *Geoffroea spinosa*, *Bursera graveolens*, *Piscidia carthagenensis*, *Ceiba trichistandra*, and *Loxopterigyum huasango*), while another group shows a northern habitat displacement migration at 0.7 km/year (i.e., *Guazuma ulmifolia*, *handroanthus chrysanthus*, *Prosopis juliflora*, *Caesalpinia glabrata*, *Cordia macrantha*, *Colicodendrum scabridum*, *Coccoloba ruiziana*, and *Cavanillesia platanifolia*). Overall, the core distributional shift directions indicate that the pathways for habitat displacements are species-specific and display divergent patterns.

## Discussion

Species distribution models are useful tools for conservation planning, resource management, and policy development [61], although the implementation and interpretation of these models may present challenges [62, 63]. Our study represents the first attempt to use exploratory modeling in a dry forest flora community to compare deforestation and climate change threats at the species and community levels.

In our models, the precipitation of the driest quarter and soil class were the two variables that exerted the largest influence on the distribution of dry forest tree species. Precipitation during the dry season has been suggested as the most critical element for determining vegetation patterns [64] because water remains a critical factor driving basic physiological processes of tropical trees [65]. Conversely, soil characteristics differentiate seasonally dry forests from savannas, even under similar climate regimes [66] and have been shown to affect the vegetation structure in Northern Peruvian dry forests [67]. In the context of major environmental processes, our study region represents typical characteristics for tropical dry forests.

With regards to model validation, we focused on the operational capability of the outputs rather than testing their inferences about the real system (e.g., Rykiel [68]). Therefore, validations used AUC values, which also show a degree of correlation with other model performance metrics [69]. In all cases, we obtained high AUC values with considerably low replicate variation (< 0.07 SD), which is half of the recommended value by other studies for “accurate and stable” models that meet practical needs [70,71]. Furthermore, as additional support to our models, the highest concentration (i.e., stacked distribution) of all 17 species agrees with the southwestern Ecuador habitat description (as depicted by Aguirre et al. [36]), providing a more practical validation of model accuracy.

According to a study based on satellite and aerial photography data, deforestation in southwestern Ecuador dry deciduous forests was approximately 29 and 57 km^2^/year from 1976 to 1989 and 1989 to 2008, respectively [17]. However, official reports indicate a 15 km^2^/year mean annual deforestation rate between 2008 and 2014 across the study area (i.e., environmental space used during modeling) [14]. According to our species distribution models, the average deforestation loss for the 17 tree species reached 71 km^2^/year (± 43.9 SD). Our results support previous direct and indirect measurements that identified deforestation as the greatest threat to dry forest ecosystems in Ecuador [6,17]. Conversely, our area loss calculations resulting from climate change projections reached 21 km^2^/year (± 26.4 SD) for the same tree species without accounting for potential distribution gain (i.e., potential new favorable areas). For instance, the difference in area-changes attributed to deforestation and climate remained statistically significant when we limited the comparison to potential area loss (excluding area gain) from the future climate scenario. Area gain can be disregarded owing to low regeneration processes and conflicting land uses that might strongly inhibit natural succession.

In summary, in terms of relative area change, deforestation and climate change represented average annual area reductions of 1.4% and 0.6%, respectively. Thus, in terms of spatial coverage and effect severity, deforestation may pose a higher threat to species distributions than climate change. However, the spatial disaggregation of both processes indicates that climate change may affect a greater number of forest areas, which are not subject to conversion.

Moreover, our species overlap results in the future climate scenario (Figure D in S1 Appendix) indicate reductions in high overlap (13–17 species) areas and expansions in low and medium overlap areas (1–6 and 7–12 species), in particular, at elevations below 1000 m in southwestern Ecuador. The apparent progression into the lowlands can be explained by the future increase in the precipitation predictor variables (e.g., bio16 and bio17), considering that other climate models also predict a precipitation increment in this area [20,22]. Finally, the magnitude and direction of the core distributional shifts differ by threat and species; this fact supports the notion of climate change pushing towards novel species compositions [72,73]. One explanation for the observed divergent directions of deforestation and climate change may be that each threat has different underlying mechanisms; deforestation pressure is determined by land use, access roads, and other density-dependent human factors, while climate change pressure is estimated solely by changes in environmental variables and responses will depend on species-specific evolutionary histories and physiological requirements. Furthermore, northward or southward species migrations might be the only possible routes for species in southwestern Ecuador given the altitude in these paths gradually increases, while two major geographic barriers, the Andes and the Pacific Ocean, lie to its East and West, respectively.

Furthermore, as our projections predict range expansions for some species and range contractions for others (in addition to the range shifts induced by deforestation and climate), tree species distributions are potentially susceptible to a myriad of unpredictable community-level effects. Thus, as an alternative to evaluating the effects of species re-assemblage on the dry forests, we propose a functional traits approach that emphasizes at the level of response traits and effect traits. As derived by Suding et al. [29], when the geographical displacement of each response group contains species from each effect group, ecosystem function resilience is expected. Conversely, the occurrence of new species assemblages under future climate scenarios may have consequences for the provision of supporting services, as well as regulating services. For instance, Sakschewski et al. [74] provided theoretical evidence that plant trait diversity may enable large-scale ecosystems to adjust to new climate conditions through competition. Moreover, in the particular case of novel combination of species, Lugo [75] asserted that tropical novel forests might behave similarly to native forests, specifically in terms of soil protection, nutrient cycles, wildlife support, carbon storage, and watershed function maintenance. Thus, we believe that changes in species composition in the dry deciduous forests of Ecuador represent challenges for forest management and requires exploration of new strategies to maintain the long-term provision of ecosystem services.

Measures to avoid deforestation should be promoted in a deforestation hotspot in the southernmost coastal region [76] and below 1000 m of elevation according to our study. Concurrently, conservation should be encouraged in the southwestern border with Peru [77], where our results predict the highest concentration of species of this forest type. Further research must incorporate deforestation data and modeling scenarios for northern Peru where the underlying causes for deforestation are similar [18,78], and social processes such as demographics, economics, and policy play an important role [76]. Therefore, strategies to avoid deforestation and promote conservation include expanding protected areas, biological corridors, species-specific timber use regulations and flagship species valorizations (e.g., *Cavanillesia platanifolia*). However, regardless of the conservation strategy, successfully achieving these goals will require both private landowner and local community participation [79].

Less than 10% of the original extent of dry deciduous forests remains in the neotropics [3] and only 2.3% is under conservation in Ecuador [6]. Therefore, it is likely that the Aichi target Nr. 11, which aims to protect 17% of terrestrial land by 2020 [80], may not be achieved for this biome in Ecuador. The results of this study can be used as an additional resource for decision-making regarding the improvement or expansion of existing protected areas and biological corridors. For instance, there is almost no mid to high altitude (100–1000 m) land under conservation in southwestern Ecuador with the exception of scarce private reserves such as La Ceiba and Laipuna. Our study highlights conservation area weaknesses in southern Ecuador, in agreement with Cuesta et al. [77]

If the protection of individual species is prioritized, attention should be given to *Albizia multiflora*, *Ceiba trichistandra*, and *Cochlospermum vitifolium* because they are heavily threatened by both deforestation and climate change. Moreover, because these species can be used for timber and in agroforestry systems [43], we recommend a special focus on sustainable management practices and gene pool conservation. With regard to species heavily threatened by deforestation and with high and valuable usage as timber and wood products (i.e., *Cordia macrantha*, *Guazuma ulmifolia*, *Handroanthus chrysanthus* and *Prosopis juliflora*), we recommend species-specific measures for their conservation, such as potential genecological zone identification, potential subpopulation evaluation, and gene pool variability tests. In combination with enrichment planting efforts, additional measures for sustainable management of these species include the establishment of seed orchards and in vitro propagation.

## Conclusion

Using presence-only modeling and native forest masks from the Ecuadorian Ministry of Environment, we obtained approximations of characteristic tree species distributions in the dry deciduous forest of southwestern Ecuador, which are threatened by deforestation and climate change. Our estimates indicate that deforestation affects more spatial range than climate change, even under an extreme climate change scenario. Despite this result, climate change may cause additional stress at the species and community levels. Special attention to *Albizia multiflora*, *Ceiba trichistandra*, and *Cochlospermum vitifolium* populations may be required because our results reveal that these species are vulnerable to both deforestation and climate change. The diverging displacement shifts of a large number of species may indicate the commencement of plant community disaggregation and a transition toward novel ecosystems. However, further research is required to discern the effects on the synecology, resilience, and ecosystem services of dry forest ecosystems.

Our results indicate that the effects of climate change result bigger in terms of distributional shifts but that deforestation affects more surface area. Therefore, as climate change adaptation and deforestation reduction measures in Ecuador likely will not match spatially, deforestation reduction should be prioritized over climate change adaptation. Similar cases of habitat loss due to these two threats may be occurring in other ecosystems in the Tropics, where annual deforestation rates are considerably far more severe [81] and novel species assemblages (i.e., redistribution) are not sufficiently considered in policies and international agreements [82].

## Supporting information

S1 Dataset Occurrence records. (XLS)

S1 Appendix Additional information. (DOCX)

## References

[1] Sanchez-Azofeifa GA, Quesada M, Rodriguez JP, Nassar JM, Stoner KE, Castillo A, et al Research Priorities for Neotropical Dry Forests. Biotropica. 2005; 37(4):477–85.

[2] Pennington RT, Lewis GP, Ratter JA. An Overview of the Plant Diversity, Biogeography and Conservation of Neotropical Savannas and Seasonally Dry Forests Neotropical Savannas and Seasonally Dry Forests: Plant Diversity, Biogeography and Conservation 2006 1–29 p.

[3] Banda R K, Delgado-Salinas A, Dexter KG, Linares-Palomino R, Oliveira-Filho A, Prado D, et al Plant diversity patterns in neotropical dry forests and their conservation implications. Science. 2016; 353(6306):1383–7. doi: 10.1126/science.aaf5080 27708031 10.1126/science.aaf5080

[4] Miles L, Newton AC, DeFries RS, Ravilious C, May I, Blyth S, et al A global overview of the conservation status of tropical dry forests. J Biogeogr. 2006; 33:491–505.

[5] Geist HJ, Lambin EF. Dynamic Causal Patterns of Desertification. Bioscience. 2004; 54(9):817–29.

[6] Portillo-Quintero CA, Sanchez-Azofeifa GA. Extent and conservation of tropical dry forests in the Americas. Biol Conserv. 2010; 143:144–55.

[7] Sanchez-Azofeifa GA, Calvo-Alvarado J, Espírito-Santo MM Do, Fernandes GW, Powers JS, Quesada M. Tropical Dry Forests in the Americas: The Tropi-Dry Endeavor In: Sanchez-Azofeifa GA, Powers JS, Fernandez GW, Quesada M, editors. Tropical Dry Forests in the Americas 2013 p. 1–15.

[8] Thomas CD, Cameron A, Green RE, Bakkenes M, Beaumont LJ, Collingham YC, et al Extinction risk from climate change. Nature. 2004; 427:145–8. doi: 10.1038/nature02121 14712274 10.1038/nature02121

[9] Sekercioglu CH, Schneider SH, Fay JP, Loarie SR. Climate change, elevational range shifts, and bird extinctions. Conserv Biol. 2008; 22(1):140–50. doi: 10.1111/j.1523-1739.2007.00852.x 18254859 10.1111/j.1523-1739.2007.00852.x

[10] Butchart SHM, Walpole M, Collen B, van Strien A, Scharlemann JPW, Almond REA, et al Global Biodiversity: Indicators of Recent Declines. Science. 2010; 328:1164–8. doi: 10.1126/science.1187512 20430971 10.1126/science.1187512

[11] Reid W, Miller K. Keeping options alive The scientific basis for conserving biodiversity. World Resources Institure, a center for policy research 1989. 130 p.

[12] Secretariat of the Convention on Biological Diversity. Global biodiversity outlook 1: Annexes [Internet]. 2001 [cited 2017 Jan 28]. Available from: https://www.cbd.int/gbo1/annex.shtml

[13] Ministerio de Ambiente del Ecuador. Mapa interactivo ambiental [Internet]. 2015 [cited 2016 Aug 20]. Available from: http://mapainteractivo.ambiente.gob.ec/portal/

[14] Ministerio de Ambiente del Ecuador. Estadísticas del Patrimonio Natural: Datos de bosques, ecosistemas, especies, carbono y deforestación del Ecuador continental. 2015 Unidad de Procesamiento de Información y Geomática.

[15] Rodriguéz-Mahecha JV, Salaman P, Jorgensen P, Consiglio T, Suárez L, Arjona F, et al Tumbes -Chocó -Magdalena In: Mittermeier RA, Robles-Gil P, Hoffmann M, Pilgrim J, Brooks T, Mittermeier CG, et al., editors. Hotspots revisited: Earth´s Biologically Richest and Most Endangered Terrestrial Ecoregions. 1st ed. Mexico city: CEMEX S.A.; 2004 p. 80–4.

[16] World Wildlife Fund. Tropical and subtropical dry broadleaf forests: Southwestern Ecuador and Northwestern Peru [Internet]. 2001 [cited 2016 Sep 28]. Available from: http://www.worldwildlife.org/ecoregions/nt0232

[17] Tapia-Armijos MF, Homeier J, Espinosa CI, Leuschner C, De La Cruz M. Deforestation and forest fragmentation in south Ecuador since the 1970s - Losing a hotspot of biodiversity. PLoS One. 2015; 10(9):1–18. 10.1371/journal.pone.0133701 PMC4557835 26332681

[18] Wunder S. The economics of deforestation The example of Ecuador. New York: Palgrave Macmillan; 2000. 262 p.

[19] Ochoa PA, Fries A, Mejía D, Burneo JI, Ruíz-Sinoga JD, Cerdà A. Effects of climate, land cover and topography on soil erosion risk in a semiarid basin of the Andes. Catena. Elsevier B.V.; 2016; 140:31–42.

[20] Magrin GO, Marengo JA, Boulanger J-P, Buckeridge MS, Castellanos E, Poveda G, et al Central and South America In: Climate Change 2014: Impacts, Adaptation, and Vulnerability. Part B: Regional Aspects. Contribution of Working Group II to the Fifth Assessment Report of the Intergovernmental Panel on Climate Change In: Barros VR, Field CB, Dokken DJ, Mastrandrea MD, Mach KJ, Bilir TE, et al, editors. Climate Change 2014: Impacts, Adaptation, Vulnerability Part B: Regional Aspects Contribution of Working Group II to the Fifth Assessment Report of the Intergovernmental Panel on Climate Change. Cambridge, United Kingdom and New York, USA: Cambridge University Press; 2014 p. 1499–566.

[21] Villacís M. Ressources en eau glaciaire dans les Andes d’Equateur en relation avec les variations du climat: Le cas du volcan Antisana. Université Montpellier II; 2008.

[22] Peters T, Drobnik T, Meyer H, Rankl M, Richter M, Rollenbeck R, et al Environmental changes afecting the Andes of Ecuador In: Bendix J, editor. Ecosystem Services, Biodiversity and Environmental Change in a Tropical Mountain Ecosystem of South Ecuador. Berlin: Heidelberg; 2013 p. 19–29.

[23] Giorgi F, Diffenbaugh N. Developing regional climate change scenarios for use in assessment of effects on human health and disease. Clim Res. 2008; 36:141–51.

[24] Marengo JA, Ambrizzi T, da Rocha RP, Alvez LM, Cuadra SV, Valverde MC, et al Future change of climate in South America in the late twenty-first century: intercomparison of scenarios from three regional climate models. Clim Dyn. 2010; 35:1073–97.

[25] Condom T, Escobar M, Purkey D, Pouget JC, Suarez W, Ramos C, et al Simulating the implications of glaciers’ retreat for water management: a case study in the Rio Santa basin, Peru. Water Int. 2012; 37(4):442–59.

[26] Feeley KJ, Silman MR, Bush MB, Farfan W, Cabrera KG, Malhi Y, et al Upslope migration of Andean trees. J Biogeogr. 2011; 38(4):783–91.

[27] Rehm EM, Feeley KJ. Forest patches and the upward migration of timberline in the southern Peruvian Andes. For Ecol Manage. Elsevier B.V.; 2013; 305:204–11.

[28] Chen I, Hill JK, Ohlemüller R, Roy DB, Thomas CD. Rapid range shifts of species of climate warming. Science. 2011; 333:1024–6. doi: 10.1126/science.1206432 21852500 10.1126/science.1206432

[29] Suding KN, Lavorel S, Chapin FS, Cornelissen JHC, Díaz S, Garnier E, et al Scaling environmental change through the community-level: A trait-based response-and-effect framework for plants. Glob Chang Biol. 2008; 14(5):1125–40.

[30] Sierra R. Propuesta preliminar de un sistema de clasificación de vegetación para el Ecuador Continental. Quito—Ecuador; 1999. 174 p.

[31] Lozano PE. Los tipos de bosque en el sur del Ecuador In: Aguirre Z, Madsen JE, Cotton E, Balslev H, editors. Estudios sobre los recursos vegetales en las provincias El Oro, Loja y Zamora-Chinchipe. Botánica Austroecuatoriana; 2002 p. 29–49.

[32] Olson DM, Dinerstein E. The Global 200: Priority ecoregions for global conservation. Ann Missouri Bot Gard. 2002; 89(2):199–224.

[33] Aguirre Z, Kvist LP, Sánchez T O. Bosques secos en Ecuador y su diversidad In: Moraes M, Öllgaard LP, Kvist LP, editors. Botánica Económica de los Andes Centrales. La Paz: Universidad Mayor de San Andrés; 2006 p. 162–87.

[34] Sáenz M, Onofa Á. Indicadores de Biodiversidad para Uso Nacional. Reporte de los ecosistemas terrestres ecuatorianos. 2005.

[35] Báez S, Salgado S, Santiana J, Cuesta F, Peralvo M, Galeas R, et al Propuesta Metodológica para la Representación Cartográfica de los Ecosistemas del Ecuador Continental. Quito; 2010.

[36] Aguirre Z, Loja A, Solano C, Aguirre N. Especies forestales más aprovechadas en la región sur del Ecuador. Universidad Nacional de Loja; 2015. 128 p.

[37] Hannah L, Lohse D, Hutchinson C, Carr J, Lankerani A. A preliminary inventory of human disturbance of world ecosystems. Ambio. 1994; 23(4/5):246–50.

[38] Sierra R, Campos F, Chamberlin J. Assessing biodiversity conservation priorities: ecosystem risk and representativeness in continental Ecuador. Landsc Urban Plan. 2002; 59(2):95–110.

[39] Aguirre Z, Lars P. Floristic composition and conservation status of the dry forests in Ecuador. Lyonia. 2005; 8(2).

[40] Parker TA, Carr JL. Status of forest remnants in the Cordillera de la Costa and adjacent areas of southwestern Ecuador. 1992.

[41] Espinosa CI, De La Cruz M, Luzuriaga L, Escudero A. Bosques tropicales secos de la región Pacífico Ecuatorial: diversidad, estructura, funcionamiento e implicaciones para la conservación. Ecosistemas. 2012; 21(1–2):167–79.

[42] Jørgensen PM, León-Yánez S. Catalogue of the Vascular Plants of Ecuador. Monogr Syst Bot Missouri Bot Gard. 1999; 75(i–viii):1–1182.

[43] Aguirre Z. Especies forestales de los bosques secos del Ecuador. Guía dendrológica para su identificación y caracterización. Quito—Ecuador: Proyecto Manejo Forestal Sostenible ante el Cambio Climático. MAE/FAO—Finlandia; 2012. 130 p.

[44] Linares-Palomino R, Kvist LP, Aguirre Z, Gonzales-Inca C. Diversity and endemism of woody plant species in the Equatorial Pacific seasonally dry forests. Biodivers Conserv. 2010; 19(1):169–85.

[45] Global Biodiversity Information Facility. GBIF Occurrence Download [Internet]. 2016 [cited 2016 Aug 5]. Available from: http://doi.org/10.15468/dl.kdccxu

[46] The Plant List. Version 1.1 Published on the Internet [Internet]. 2013 [cited 2017 Jan 21]. Available from: http://www.theplantlist.org

[47] Smith AB. elimCellDups: Eliminate duplicate points in a raster cell [Internet]. 2012 [cited 2016 Dec 12]. Available from: http://www.earthskysea.org/r-code/

[48] Hijmans RJ, Cameron SE, Parra JL, Jones PG, Jarvis A. Very high resolution interpolated climate surfaces for global land areas. Int J Climatol. 2005; 25:1965–78.

[49] Hengl T, Mendes de Jesus J, MacMillan RA, Batjes NH, Heuvelink GBM, Ribeiro E, et al SoilGrids1km—Global Soil Information Based on Automated Mapping. PLoS One. 2014; 9(8):e105992 doi: 10.1371/journal.pone.0105992 25171179 10.1371/journal.pone.0105992 PMC4149475

[50] Hengl T, Mendes De Jesus J, Heuvelink GBM, Gonzalez MR, Kilibarda M, Blagotí A, et al SoilGrids250m: Global gridded soil information based on machine learning. PLoS One. 2017; 12(2). 10.1371/journal.pone.0169748 PMC5313206 28207752

[51] Shangguan W, Hengl T, Mendes de Jesus J, Yuan H, Dai Y. Mapping the global depth to bedrock for land surface modeling. J Adv Model Earth Syst. 2017; 9:65–88.

[52] IPCC. Climate Change 2014: Synthesis Report. Contribution of Working Groups I, II and III to the Fifth Assessment Report of the Intergovernmental Panel on Climate Change. Geneva, Switzerland; 2015.

[53] Giovanelli JGR, de Siqueira MF, Haddad CFB, Alexandrino J. Modeling a spatially restricted distribution in the Neotropics: How the size of calibration area affects the performance of five presence-only methods. Ecol Modell. 2010; 221:215–24.

[54] Barve N, Barve V, Jiménez-Valverde A, Lira-Noriega A, Maher SP, Peterson AT, et al The crucial role of the accessible area in ecological niche modeling and species distribution modeling. Ecol Modell. 2011; 222(11):1810–9.

[55] Wisz MS, Hijmans RJ, Li J, Peterson AT, Graham CH, Guisan A. Effects of sample size on the performance of species distribution models. Divers Distrib. 2008; 14(5):763–73.

[56] Merow C, Smith MJ, Silander J a. A practical guide to MaxEnt for modeling species’ distributions: What it does, and why inputs and settings matter. Ecography (Cop). 2013; 36(10):1058–69.

[57] Lobo JM, Jiménez-valverde A, Real R. AUC: A misleading measure of the performance of predictive distribution models. Glob Ecol Biogeogr. 2007; 17(2):145–51.

[58] Peterson AT, Soberón J, Pearson RG, Anderson RP, Martinez-Meyer E, Nakamura M, et al Ecological niches and geographic distributions. Princeton University Press. Princeton, New Jersey: Princeton University Press; 2011. 314 p.

[59] Jung M. LecoS–A python plugin for automated landscape ecology analysis. Ecol Inform. 2016; 31:18–21.

[60] Brown JL. SDMtoolbox: A python-based GIS toolkit for landscape genetic, biogeographic and species distribution model analyses. Methods Ecol Evol. 2014; 5(7):694–700. 10.7717/peerj.4095 PMC5721907 29230356

[61] Franklin J. Mapping species distributions: spatial inference and prediction. Cambridge, U.K.: Cambridge University Press; 2010. 319 p.

[62] Franklin J. Species distribution models in conservation biogeography: Developments and challenges. Divers Distrib. 2013; 19(10):1217–23.

[63] Booth TH. Assessing species climatic requirements beyond the realized niche: some lessons mainly from tree species distribution modelling. Clim Change. 2017; 145(3–4):259–271.

[64] Malhi Y, Roberts JT, Betts RA, Killeen TJ, Li W, Nobre CA. Climate change, deforestation, and the fate of the Amazon. Science. 2008; 319(5860):169–72. doi: 10.1126/science.1146961 18048654 10.1126/science.1146961

[65] van der Sleen P, Groenendijk P, Vlam M, Anten NPR, Boom A, Bongers F, et al No growth stimulation of tropical trees by 150 years of CO2 fertilization but water-use efficiency increased. Nat Geosci. 2015; 8(1):24–8.

[66] Sarmiento G. A conceptual model relating environmental factors and vegetation formations in the lowlands of tropical South America In: Furley PA, Proctor J, Ratter JA, editors. Nature and dynamics of forest-savanna boundaries. London, UK: Chapman & Hall; 1992 p. 583–601.

[67] Muenchow J, von Wehrden H, Rodríguez EF, Rodríguez RA, Bayer F, Richter M. Woody vegetation of a peruvian tropical dry forest along a climatic gradient depends more on soil than annual precipitation. Erdkunde. 2013; 67(3):241–8.

[68] Rykiel EJ. Testing ecological models: The meaning of validation. Ecol Modell. 1996; 90(3):229–44.

[69] Allouche O, Tsoar A, Kadmon R. Assessing the accuracy of species distribution models: Prevalence, kappa and the true skill statistic (TSS). J Appl Ecol. 2006; 43(6):1223–32.

[70] Ramírez-Villegas J, Khoury C, Jarvis A, Debouck DG, Guarino L. A Gap analysis methodology for collecting crop genepools: A case study with Phaseolus beans. PLoS One. 2010; 5(10) e13497 doi: 10.1371/journal.pone.0013497 20976009 10.1371/journal.pone.0013497 PMC2958131

[71] Cobben MMP, van Treuren R, Castañeda-Álvarez NP, Khoury CK, Kik C, van Hintum TJL. Robustness and accuracy of Maxent niche modelling for Lactuca species distributions in light of collecting expeditions. Plant Genet Resour. 2015; 13(2):153–61.

[72] Hobbs RJ, Higgs ES, Hall CM. Novel Ecosystems: Intervening in the New Ecological World Order. Novel Ecosystems: Intervening in the New Ecological World Order. 2013. 1–368 p.

[73] Ordonez A, Williams JW, Svenning J-C. Mapping climatic mechanisms likely to favour the emergence of novel communities. Nat Clim Chang. 2016; 6(12):1104–9.

[74] Sakschewski B, von Bloh W, Boit A, Poorter L, Peña-Claros M, Heinke J, et al Resilience of Amazon forests emerges from plant trait diversity. Nat Clim Chang. 2016; 6(11):1032–6.

[75] Lugo AE. The emerging era of novel tropical forests. Biotropica. 2009; 41(5):589–91.

[76] Sierra R. Patrones y factores de deforestación en el Ecuador continental, 1990–2010. Y un acercamiento a los próximos 10 años. Quito—Ecuador: Conservation International, Forest Trends; 2013. 51 p.

[77] Cuesta F, Peralvo M, Merino-Viteri A, Bustamante M, Baquero F, Freile JF, et al Priority areas for biodiversity conservation in mainland Ecuador. Neotrop Biodivers. Taylor & Francis; 2017; 3(1):93–106.

[78] Linares-palomino R. Los Bosques Tropicales Estacionalmente Secos: II. Fitogeografía y Composición florística. Arnaldoa. 2004; 11(1):103–38.

[79] Blackie R, Baldauf C, Gautier D, Gumbo D, Kassa H, Parthasarathy N, et al. Tropical dry forests: The state of global knowledge and recommendations for future research. Discussion Paper. Bogor, Indonesia; 2014.

[80] Convention on Biological Diversity. Quick guide to the Aichi Biodiversity Targets: Protected areas increased and improved, TARGET 11—Technical Rationale extended [Internet]. 2011 [cited 2017 May 4]. Available from: https://www.cbd.int/doc/strategic-plan/targets/T11-quick-guide-en.pdf

[81] Hansen MC, Potapov P V., Moore R, Hancher M, Turubanova SA, Tyukavina A, et al High-Resolution Global Maps of 21st-Century Forest Cover Change. Science. 2013; 342(6160):850–3. doi: 10.1126/science.1244693 24233722 10.1126/science.1244693

[82] Pecl GT, Araújo MB, Bell JD, Blanchard J, Bonebrake TC, Chen I-C, et al Biodiversity redistribution under climate change: Impacts on ecosystems and human well-being. Science. 2017; 355(6332):eaai9214 doi: 10.1126/science.aai9214 28360268 10.1126/science.aai9214

