# Correction: Climate change versus deforestation: Implications for tree species distribution in the dry forests of southern Ecuador

**DOI:** 10.1371/journal.pone.0195851

**Published:** 2018-04-09

**Authors:** Carlos E. Manchego, Patrick Hildebrandt, Jorge Cueva, Carlos Iván Espinosa, Bernd Stimm, Sven Günter

There is an error in the first sentence of the second paragraph in the Introduction section. The correct sentence is: Ecuador, one of the ten most biodiversity-rich nations in the world [11, 12], has one-sixth of its territory covered by deciduous and semi-deciduous forests [13] with a reported national deforestation rate of approximately 475 km^2^/year during 2008–2014 [14].
