# Supplementary material for: Climate change versus deforestation: Implications for tree species distribution in the dry forests of southern Ecuador
Source: PLoS One. 2017 Dec 21;12(12):e0190092. doi: 10.1371/journal.pone.0190092 (PMC5739474; doi:10.1371/journal.pone.0190092)
Supplement: S1 Appendix — (DOCX) [file pone.0190092.s002.docx]

**Table A.** **Average response curves of predictor variables used during modeling:** bio1 = annual mean temperature, bio2 = mean diurnal range, bio15 = precipitation seasonality, bio16 = precipitation of wettest quarter, bio17 = precipitation of driest quarter, used in conjunction with soil classification, depth to bedrock and soil organic content. Omitted variables were removed due to correlation coefficient >0.8. Values in percentages represent estimates of relative contributions of the environmental variable to the overall Maxent model. Numbers in bold show the highest contribution. Pages 1 and 2.

| Species | bio01 | bio02 | bio15 | bio16 | bio17 | Soil class | Soil depth | Soil org. cont. |
| --- | --- | --- | --- | --- | --- | --- | --- | --- |
| *Albizia multiflora* | 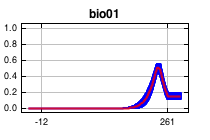5.4% | 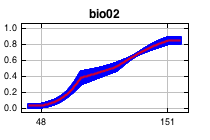4.5% | 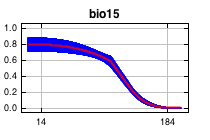3.7% | 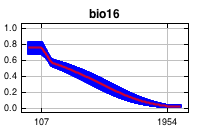1.1% | 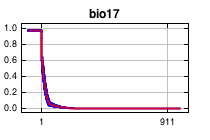**50.4%** | 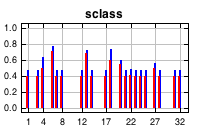13% | 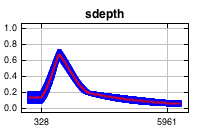12.6% | 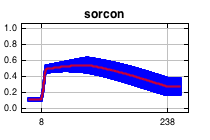9.3% |
| *Bursera graveolens* | 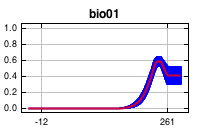5.6% | 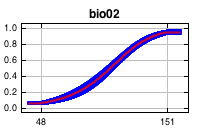8.5% | 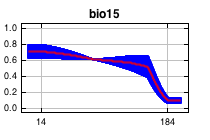1.6% | 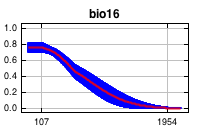2.5% | 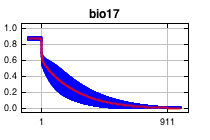**38.6%** | 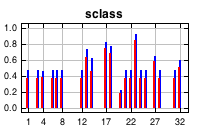35% | 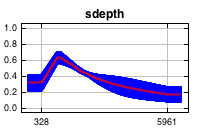5.7% | 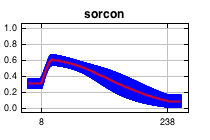2.5% |
| *Caesalpinia glabrata* | 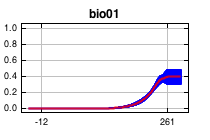2.2% | 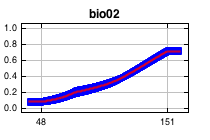3.5% | 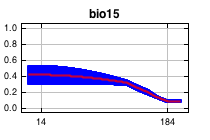2.1% | 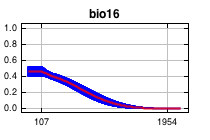1.3% | 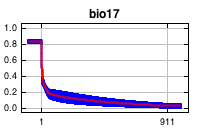**52.7%** | 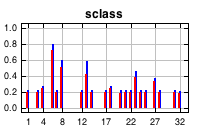23.5% | 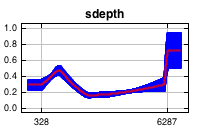5.9% | 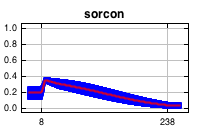8.8% |
| *Cavanillesia platanifolia* | 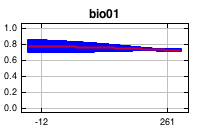1.8% | 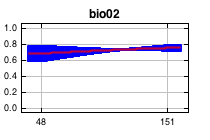0.7% | 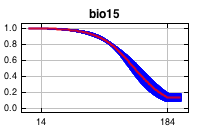4.4% | 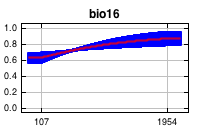0.2% | 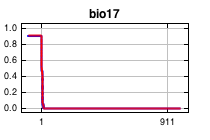**68.7%** | 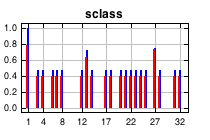24.1% | 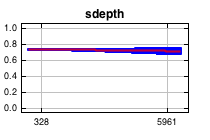0% | 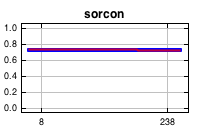0% |
| *Ceiba trichistandra* | 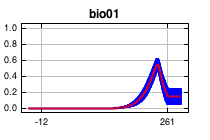4.3% | 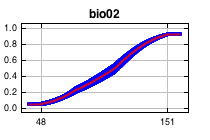7.6% | 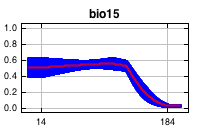3% | 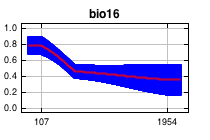0.5% | 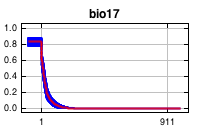**36.7%** | 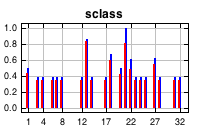26.3% | 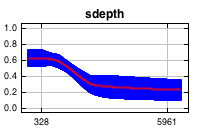7.1% | 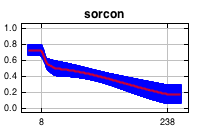14.5% |
| *Chloroleucon mangense* | 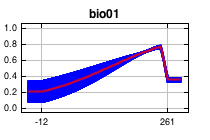1.6% | 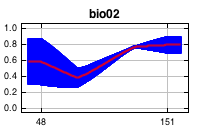13% | 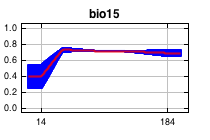0.2% | 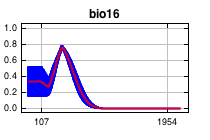13.7% | 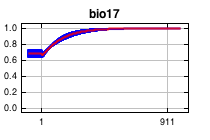2.8% | 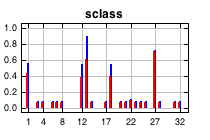**62.9%** | 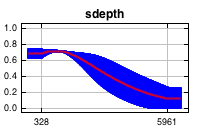4.3% | 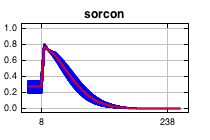1.7% |
| *Cordia macrantha* | 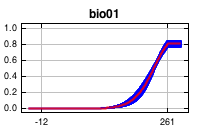23.6% | 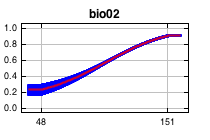10.3% | 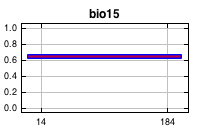0% | 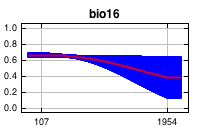3.9% | 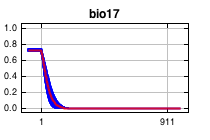22.3% | 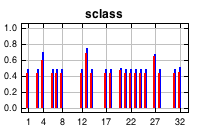**33.1%** | 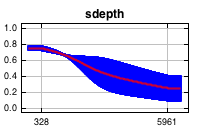6% | 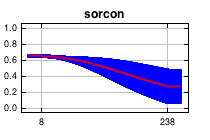0.7% |
| *Coccoloba ruiziana* | 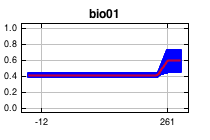0.3% | 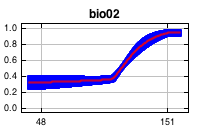7.8% | 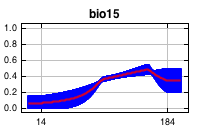3.8% | 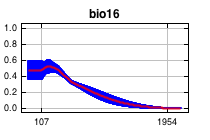3.3% | 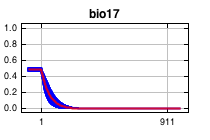**44.1%** | 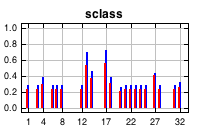22.8% | 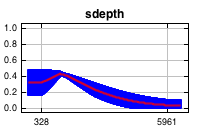10.1% | 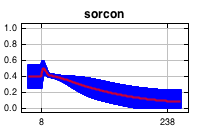7.8% |
| *Colicodendron scabridum* | 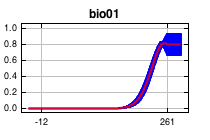8.7% | 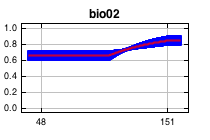0.7% | 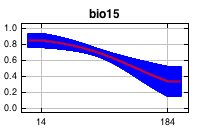0.5% | 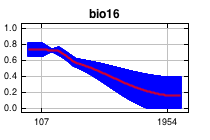3.2% | 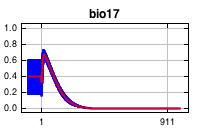14.2% | 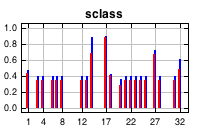**28.4%** | 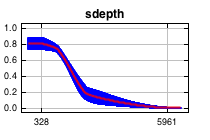17.8% | 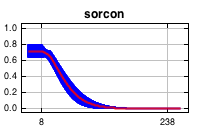26.6% |
| *Cochlospermum vitifolium* | 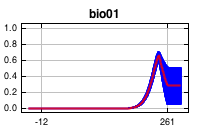8.4% | 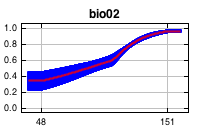8.8% | 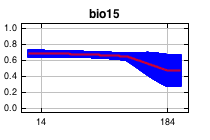2.9% | 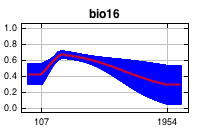1.7% | 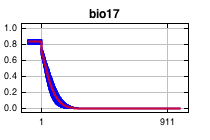**35.4%** | 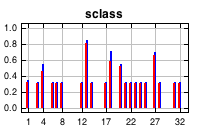30.1% | 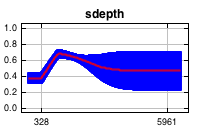2.5% | 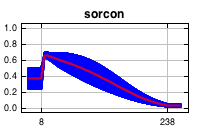10.3% |
| *Erythrina velutina* | 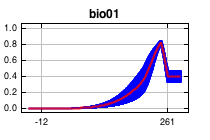9.4% | 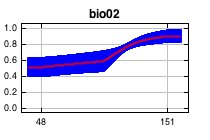6.1% | 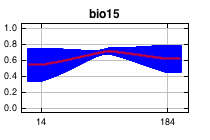4% | 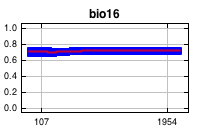0.1% | 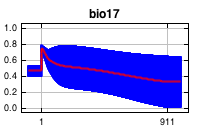23.3% | 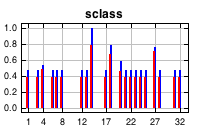**35.2%** | 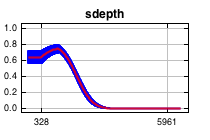20.3% | 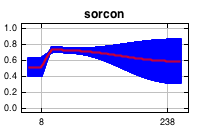1.6% |
| *Geoffroea spinosa* | 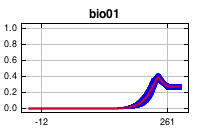3% | 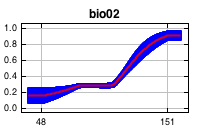13% | 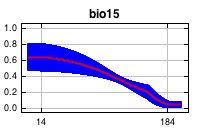3.1% | 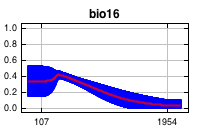1.8% | 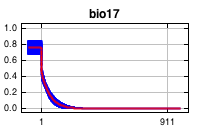**31.2%** | 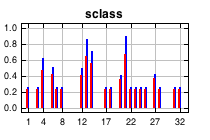20.2% | 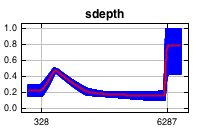4.7% | 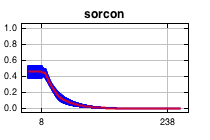23.1% |
| *Guazuma ulmifolia* | 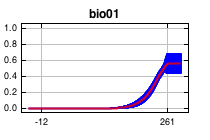12% | 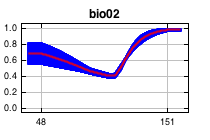4% | 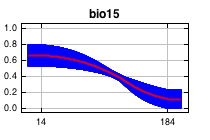7.4% | 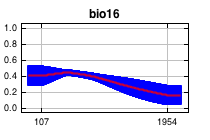2% | **48.1%** | 15.4% | 2.8% | 8.3% |
| *Handroanthus chrysanthus* | 1.8% | 1.5% | 8.7% | 4.8% | **51.7%** | 13.7% | 7.4% | 10.5% |
| *Loxopterygium huasango* | 4.5% | 9% | 0.8% | 0% | **39.4%** | 31.1% | 5.9% | 9.4% |
| *Piscidia carthagenensis* | 2.5% | 8.6% | 2.3% | 5.2% | 31.4% | **47.1%** | 2.7% | 0.2% |
| *Prosopis juliflora* | 7% | 2.1% | 1.4% | 16% | **61.8%** | 7.8% | 0.9% | 2.9% |

**Table B. Comparison of the threats of deforestation and climate change in the distribution 17 characteristic tree species of the Ecuadorian dry deciduous forest.** Gridded area represents the extent of environmental space used during modeling. Images in the right side emphasize the location of southwestern Ecuador. Colors represent the areas of potential distribution under: remnant native forest by 2014 (green); areas lost to deforestation, period 2008-2014 (black); areas of remnant forest threatened by climate change, period 2014-2050 (gray). Pages 3 to 8.

| \| *Albizia multiflora* \|  \| \| --- \| --- \| \| *Bursera graveolens* \|  \| \| *Caesalpinia glabrata* \|  \| \| *Cavanillesia platanifolia* \|  \| \| *Ceiba trichistandra* \|  \| |
| --- | --- | --- | --- | --- | --- | --- | --- | --- | --- | --- |
| \| *Chloroleucon mangense* \|  \| \| --- \| --- \| \| *Cordia macrantha* \|  \| \| *Coccoloba ruiziana* \|  \| \| *Colicodendron scabridum* \|  \| \| *Cochlospermum vitifolium* \|  \| \| *Erythrina velutina* \|  \| |
| \| *Geoffroea spinosa* \|  \| \| --- \| --- \| \| *Guazuma ulmifolia* \|  \| \| *Handroanthus chrysanthus* \|  \| \| *Loxopterygium huasango* \|  \| |
| \| *Piscidia carthagenensis* \|  \| \| --- \| --- \| \| *Prosopis juliflora* \|  \| |

**Table C. Change in annual landscape statistics according to groups of species overlap.** Proportion (%) adjusted based in approximate extent of the Ecuadorian dry forest according to Portillo-Quintero & Sanchez-Azofeifa, 2010, approximately 25,000 km^2^. Number of patches shows percent change relative to of all patches.

| **Species overlap** | **Low (1 to 6 species)** | | **Moderate (7 to 12 species)** | | **High (13 to 17 species)** | |
| --- | --- | --- | --- | --- | --- | --- |
|  | *Deforestation* | *Climate* | *Deforestation* | *Climate* | *Deforestation* | *Climate* |
| Land cover km^2^ | -157.25 | 32.42 | -52.23 | 8.00 | -33.77 | -5.60 |
| Proportion % | -0.10 | 0.02 | -0.03 | 0.01 | -0.02 | 0.00 |
| Edge length km | -497.22 | 169.74 | -260.28 | 28.74 | -59.50 | -13.30 |
| Number of patches % | -0.3 | 0.4 | -2.0 | 0.01 | 0.7 | -0.05 |
| Mean patch area km^2^ | -0.02 | 0.002 | 0.002 | 0.002 | -0.07 | -0.01 |
| Greatest patch area km^2^ | -3.24 | 1.01 | -1.39 | 0.60 | -5.45 | -1.17 |
| Smallest patch area km^2^ | 0 | 0 | 0 | 0 | 0 | 0 |

**Figure D. Map of differences in the stacked distribution of 17 characteristic tree species of the Ecuadorian dry forests.** Change attributed to deforestation, 2008-2014 (a), and change attributed to future climate, 2014-2050 CCSM4.0, RCP 8.5 (b). Dashed lines represent contour lines, elevation in m a.s.l.

**Table E. Values of area loss projected according to threat.** Comparison of the effects of deforestation and climate change on species distribution, ignoring new suitable areas from the future climate model.
